# Overexpression of both platelet-derived growth factor-BB and vascular endothelial growth factor-C and its association with lymphangiogenesis in primary human non-small cell lung cancer

**DOI:** 10.1186/1746-1596-9-128

**Published:** 2014-06-27

**Authors:** Jiannan Liu, Chuanyong Liu, Liyun Qiu, Juan Li, Pei Zhang, Yuping Sun

**Affiliations:** 1 Department of Oncology, Jinan Central Hospital, Affiliated to Shandong University, No. 105.Jiefang Road, Jinan, Shandong 250013, P.R. China; 2 Department of Oncology, Yuhuangding Hospital, Yantai, Shandong 264000, P.R. China; 3 Department of Pharmacology, Jinan Central Hospital, Affiliated to Shandong University, Jinan, Shandong 250013, P.R. China

**Keywords:** Platelet-derived growth factor-BB, Vascular endothelial growth factor-C, Lymphatic micro-vessel density, Non-small cell lung cancer

## Abstract

**Abstract:**

**Background**

Metastatic spread of tumor through lymphatic vasculature is an important adverse prognostic factor in a variety of human cancer and tumor lymphangiogenesis requires the interplay of several growth factors. Platelet-derived growth factor (PDGF)-BB and vascular endothelial growth factor (VEGF)-C are two important molecules involving in tumor metastasis and lymphangiogenesis. Therefore, the aim of this study was to investigate the coexpression of PDGF-BB and VEGF-C in primary human non-small cell lung cancer (NSCLC) and its association with lymphangiogenesis.

**Methods**

Using immunohistochemical staining, PDGF-BB and VEGF-C expression were detected in 109 primary NSCLC tissues, while the lymphatic micro-vessel density (LMVD) was counted.

**Results**

Of 109 cases, PDGF-BB and VEGF-C overexpression was 66.97% (73/109) and 65.14% (71/109), respectively. 52 (47.7%) had overexpression of both PDGF-BB and VEGF-C (P + V+), 21 (19.3%) overexpression of PDGF-BB but low expression of VEGF-C (P + V-), 19(17.4%) overexpression of VEGF-C but low expression of PDGF-BB (P-V+) and 17(15.6%) low expression of both PDGF-BB and VEGF-C (P-V-). PDGF-BB expression was positively related to that of VEGF-C (r = 0.451, *p* = 0.034). LMVD in cases with P + V + was much higher than those with P-V- (*p* = 0.004). In addition, the patients with P + V + were younger and also had larger tumor size, more likely lymph node metastasis and worse histological differentiation than those with P-V-. Moreover, the overall survival (OS) of patients with P + V + was shorter than those with P-V- (*p* = 0.015).

**Conclusion**

Coexpression of both PDGF-BB and VEGF-C was associated with lymphangiogenesis and poor prognosis in NSCLC, and might play a critical role in NSCLC progression.

**Virtual Slides:**

The virtual slide(s) for this article can be found here: http://www.diagnosticpathology.diagnomx.eu/vs/2261801312571320

## Background

Lung cancer is the leading cause of tumor-related mortality throughout the world, of which 80% are non-small cell lung cancer (NSCLC). In 2008, lung cancer replaced liver cancer as the first cause of death among people with malignant tumors in China [1]. Despite all efforts in the field of early diagnosis and adjuvant therapy, the morbidity and mortality of NSCLC trend to ascend straightly [2]. One of the most important factors with direct impact on prognosis and therapeutic strategy in NSCLC is lymphatic metastasis [3,4].

Lymphangiogenesis, the formation of new lymphatic vessels, is considered to be an important process in the development of lymphatic metastasis [5]. The status of lymphangiogenesis and lymphatic vessel remodeling has been estimated by lymphatic micro-vessel density (LMVD) [6]. D2-40 is the preferred lymphatic endothelium-specific monoclonal antibody (mAb) for investigating intra-tumoral and peri-tumoral lymphatic micro-vessels [7]. Increased amount of LMVD provides more opportunities for tumor cells to disseminate to the lymph nodes. The correlation between LMVD and prognosis was confirmed in a variety of human cancer, including breast cancer, melanoma and NSCLC [8-11].

The family of VEGFs is composed of VEGF-A, VEGF-B, VEGF-C, VEGF-D, VEGF-E, VEGF-F, and placental growth factor (PlGF). VEGF-A is directly linked to angiogenesis, while VEGF-C is considered as a prime mediator of lymphangiogenesis and has been implicated in carcinogenesis and metastasis. VEGF-C is a ligand for the VEGF receptor (VEGFR)-3, a tyrosine kinase receptor that is expressed predominantly on lymphatic endothelial cells (LECs) [12,13]. It is demonstrated that VEGF-C induces lymphangiogenesis by VEGFR-3 signaling [14]. Studies showed that VEGF-C expression is associated with lymphatic invasion, LMVD, lymph node metastasis, and prognosis in some human tumors, such as breast cancer, gastric cancer and NSCLC [15-18].

Recent studies show that platelet-derived growth factors (PDGFs) also enable the process of functional lymphangiogenesis. They can connect the receptors on LECs to promote LECs’ proliferation, migration and the formation of tubular structures, which induce lymphangiogenesis [19]. PDGF family consists of five isoforms, -AA, -AB, -BB, -CC, and –DD [20]. PDGF-BB is a direct lymphangiogenic factor [21]. Emerging evidences indicate that the tight communication between vascular endothelial cells and mural cells by platelet-derived growth factor (PDGF)-BB is essential for capillary stabilization during the angiogenic process [22]. It was reported that the expression of PDGF-BB was correlated with tumor growth, lymph node metastasis and lymphatic invasion in human esophageal squmaous cell carcinomas and NSCLC [23,24].

Based on these data, PDGF-BB and VEGF-C may play an important role in the process of tumor growth and lymphangiogenesis. However, it is still unknown about the significance of combination of PDGF-BB and VEGF-C, i.e. expression of both PDGF-BB and VEGF-C, compared with only PDGF-BB, or VEGF-C expression, in NSCLC. In this study, we examined the expression of PDGF-BB and VEGF-C in primary NSCLC tissues, and investigated the clinicopathological significance of their coexpression and association with lymphangiogenesis.

## Methods

### Patients’ characteristics

Tumor specimens were obtained from 109 patients with primary NSCLC who underwent surgery at the Jinan Central Hospital Affiliated to Shandong University, China, during the period from October 2008 to September 2010. They did not receive radiation therapy or chemotherapy before biopsy or surgical resection. There were 78 men (72%) and 31 women (28%) with median age of 58 years (interquartile range: 50 ~ 65 years) at the time of diagnosis. We determined the cell differentiation degree according to the classification amended in 1999 [25] and found 81 cases of well and moderately differentiated cells and 28 cases of poorly differentiated cells. The tumors were staged according to the USA Cancer Union Guidelines [26]. 38 patients were diagnosed with early NSCLC (I-IIa) and 71 with advanced NSCLC (IIb-III). Other clinical features are summarized in Table 1. All patients were followed up for at least 3 years after surgery. The median follow-up period was 47 months (interquartile range: 42 ~ 50 months). Overall survival (OS) was calculated from the date of surgery to the last follow up. The work was conducted in accordance with the Declaration of Helsinki. Informed consent was obtained from all the patients in this study. All patients signed the informed consent for use of specimens, and the study was approved by the Institutional Review Board (Medical Ethics Committee of Jinan Central Hospital).

**Table 1:** Correlations of both PDGF-BB and VEGF-C coexpression with clinicopathological factors in primary human NSCLC

	**Factors**	**P + V+**	**P-V-**	***P1***	**P + V -**	***P2***	**P-V+**	***P3***
Gender	Male	35	13	0.476	16	0.716	14	0.847
	Female	17	4		5		5	
Age	>60 years	23	11	0.047	13	0.859	11	0.676
	≤60 years	29	6		8		8	
Histology	SQC	28	6	0.184	6	0.539	5	0.559
	ADC	24	11		15		14	
Tumor size	>5 cm	24	3	0.037	5	0.950	6	0.563
	≤5 cm	28	14		16		13	
differentiation	WD, MD	35	17	0.017	17	0.757	12	0.027
	PD	17	0		4		7	
TNM stage	I-IIa	12	8	0.113	10	0.973	8	0.765
	IIb-III	40	9		11		11	
Nodal status	Positive	29	3	0.006	5	0.471	7	0.362
	Negative	23	14		14		12	

Note: P1, P value between P + V + and P-V-; P2 , P value between P + V- and P-V-; P3, P value between P-V + and P-V-.

*Abbreviations*: WD well differentiated, MD moderately differentiated, PD poorly differentiated, ADC adenocarcinoma, SQC squamous cell carcinoma.

### Main reagents

The main reagents were anti-podoplanin mouse monoclonal antibody D2-40 (Dako Co. Denmark), anti-PDGF-BB rabbit polyclonal antibody (abcam, Cambridge, UK), Anti-VEGF-C rabbit monoclonal antibody (Beijing Zhongshan Goldenbrige Biotechnology, China), immunohistochemical SP reagent box and DAB colour reagent (Fuzhou Maixin Co. China.P.R).

### Immunohistochemistry

Immunohistochemical staining was carried out using the DAKO Envision detection kit (Dako, Carpinteria, CA, USA). In brief, paraffin-embedded tissue blocks were sectioned (4 μm-thick), dried, deparaffinized, and rehydrated. Antigen retrieval was performed in a microwave oven for 15 min in 10 mM citrate buffer (pH 6.0). For all samples, endogenous peroxidase activity was blocked with a 3% H2O2-methanol solution. The slides were blocked with 10% normal goat serum for 10 min and incubated with an appropriately diluted primary antibody mouse monoclonal antibody D2-40 (diluted 1:50), anti-PDGF-BB rabbit polyclonal antibody (diluted 1:200) or anti-VEGF-C rabbit polyclonal antibody (diluted 1:100) overnight at 4°C. The slides were then probed with an HRP-labeled polymer conjugated to an appropriate secondary antibody for 30 min. Each step was followed by washing with PBS. Each batch of staining was accompanied by positive and negative control slides. Primary human NSCLC tissues, which are demonstrated to exhibit high levels of PDGF-BB and VEGF-C protein, were used as positive controls. Normal mouse IgG substituted for primary antibody was a negative control.

### Quantitation of immunohistochemistry

Clinicopathological findings were evaluated simultaneously using a double-headed light microscope by two independent examiners in a blinded fashion and mean values were calculated. The percentage of stained cells was recorded in at least 5 fields at 400-fold magnification in randomly selected tumor areas. In tumor specimens, analysis of staining was exclusively restricted to the NSCLC cell reactions. Staining of stromal cells was not considered. Because cancer cells showed heterogeneous staining, the dominant pattern was used for scoring.

A combined scoring method that accounts for the intensity of staining as well as the percentage of cells stained was used as described previously [27]*.* The intensity of staining was graded from 0 to 3, with strong, moderate, weak, and negative staining intensities as grade 3, 2, 1, and 0, respectively. The scores indicating percentage of positive cancer cells and staining intensity were multiplied to get a weighted score for each sample. For example, a sample with 10% weak staining, 10% moderate staining, and 80% strong staining would be assigned a score of 270 (10 × 1 + 10 × 2 + 80 × 3 = 270) out of a possible score of 300. For statistical analyses, samples with weighted scores 0–100 were defined as negative, otherwise as positive.

LMVD was performed according to a modification of Weidner’s method [28]. The immunostained sections were scanned by light-microscopy at low magnification (40×) and the areas of tissue with the greatest number of distinctly highlighted microvessels (hot spots) were selected. LMVD was then determined by counting all immunostained vessels at a total magnification of (200×) from five areas for each case. Determination of the staining reaction was strictly confined to the hot spots and the mean number of the vessels in each case was evaluated.

### Statistical analysis

Data were analyzed according to the Statistical Package for Social Sciences (SPSS. 18.0 Chicago, IL, USA). Spearman’s coefficient of correlation, Chi-squared test, and two-tailed Student t test were used as appropriate. Overall survival (OS) curves were delineated by the Kaplan-Meier method and compared with log-rank test. For all tests, *p*-values less than 0.05 were considered to be significant. All *p*-values given were results of two-sided tests.

## Results

### PDGF-BB and VEGF-C coexpression in primary human NSCLC

**Figure 1:**
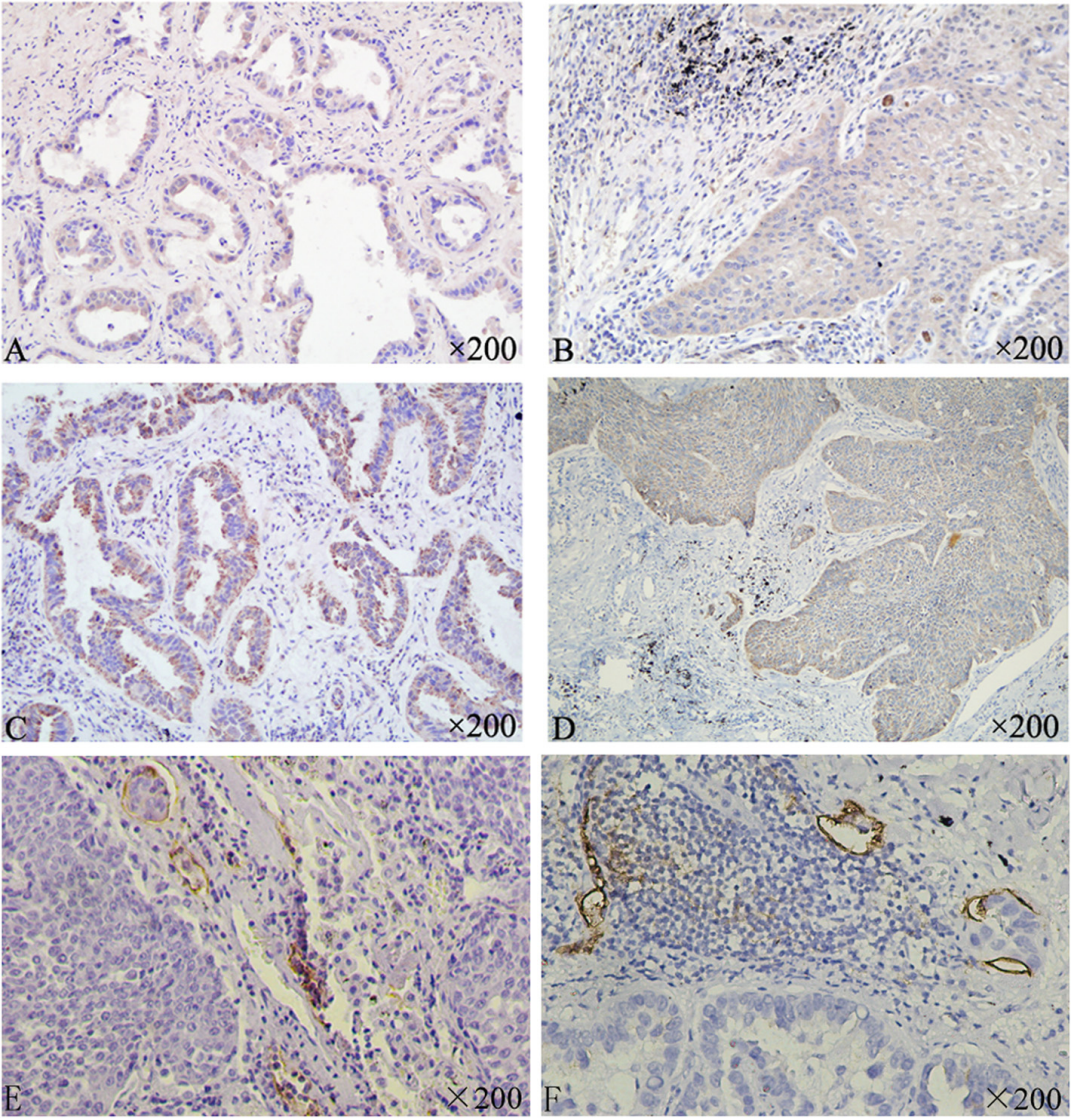
**Immunohistochemical staining for PDGF-BB, VEGF-C and D2-40 in primary NSCLC tissues (×200). A**: PDGF-BB overexpression in adenocarcinoma. **B**: PDGF-BB overexpression in squamous cell carcinoma. **C**: VEGF-C expression in adenocarcinoma. **D**: VEGF-C expression in squamous cell carcinoma. **E**: D2-40 expression in the lymphatic endothelial cells in adenocarcinoma. **F**: D2-40 expression in the lymphatic endothelial cells in squamous cell adenocarcinoma.

**Figure 2:**
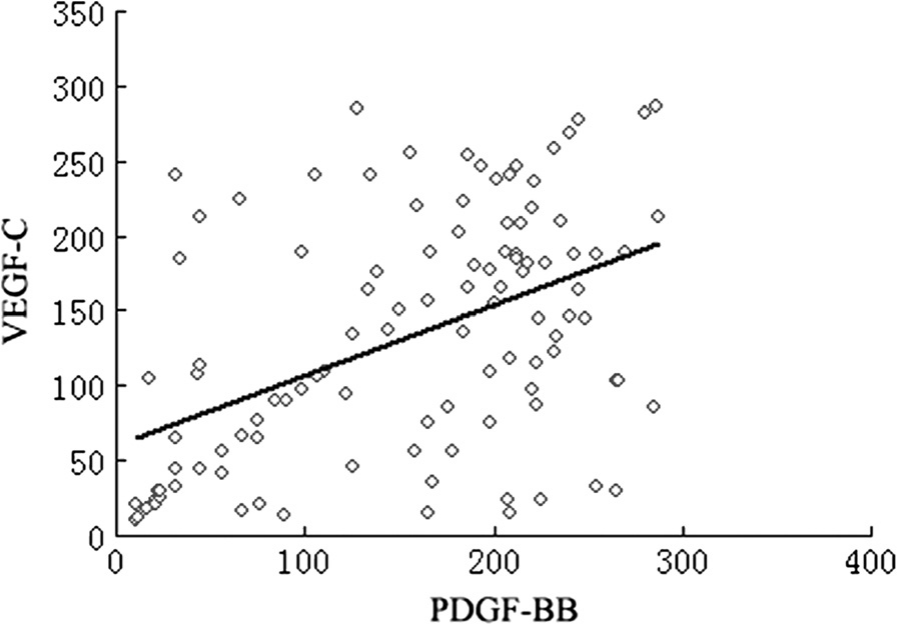
Relationship between the expression of PDGF-BB and VEGF-C in all adenocarcinoma and squamous cell carcinomas in NSCLC patients.

In primary human NSCLC tissues, PDGF-BB (Figure 1A, B) and VEGF-C (Figure 1C, D) expression were mainly present in the cytoplasm of cancer cells*.* PDGF-BB was also found on cancer cell membrane. Occasional and weak expression of PDGF-BB and VEGF-C were found in both cancer stroma and paracancerous normal tissues. Among 109 cases, PDGF-BB and VEGF-C overexpression was 66.97% (73/109) and 65.14% (71/109), respectively. A cohort of patients was classified into 4 groups according to the expression of PDGF-BB and VEGF-C in the same patient. As shown in Table 1, 47.7% (52/109) had overexpressions of both PDGF-BB and VEGF-C ( P + V+); 19.3% (21/109) had overexpression of PDGF-BB but low expression of VEGF-C (P + V-); 17.4% (19/109) patients had overexpression of VEGF-C but low expression of PDGF-BB (P-V+); 15.6% (17/109) patients had low expressions of both PDGF-BB and VEGF-C (P-V-). PDGF-BB expression had a positive correlation with that of VEGF-C (r = 0.451, *p* = 0.034) ( Figure 2).

Among 44 specimens from cases with lymph node metastasis, 29 had P + V+, 5P + V- ,7 P-V+, and 3 P-V-. There was a significant association between P + V + and lymph node metastasis (*p* = 0.006). In addition, compared with the P-V- cases, the cases with P + V + were younger (*p* = 0.047), and also had larger tumor size (*p* = 0.037) and worse histological differentiation (*p* = 0.017). While the cases with P-V + patients had worse histological differentiation (*p* = 0.027), no other clinicopathological factores were found to be related to P + V- or P-V + .

### Relationship between lymphangiogenesis and coexpression of both PDGF-BB and VEGF-C in primary human NSCLC

**Figure 3:**
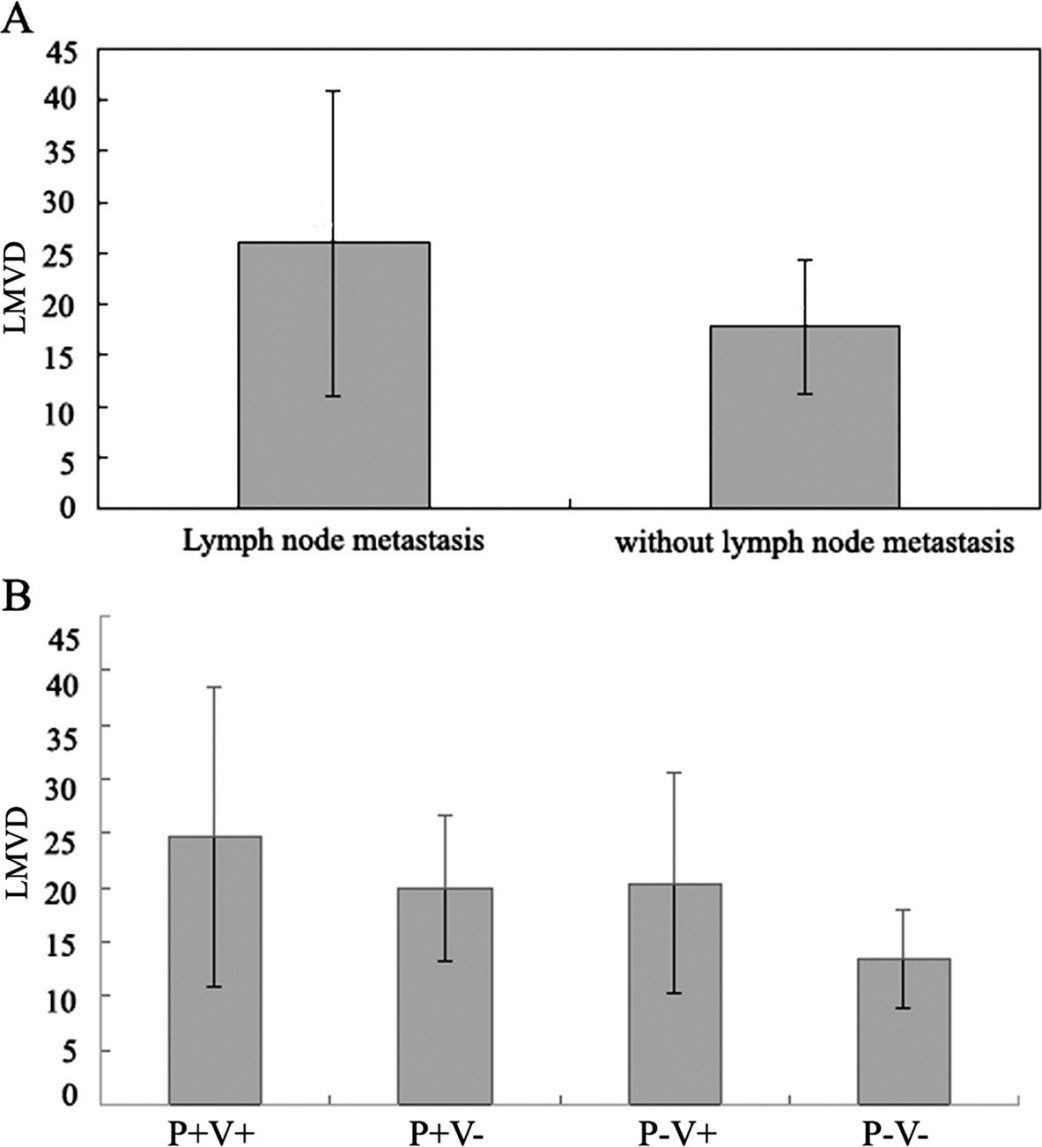
Comparison of LMVD between the patients (A) who had lymph node metastasis and who didn’t, and among the patients (B) who had P + V+, P + V-, P-V + and P-V-.

D2-40 expression was strictly present in the lymphatic endothelial cells. D2-40 positive lymphatic vessels were almost exclusively found at the tumor’s invasion front within the tumor stroma. The peri-tumoral lymphatic vessels were dilated and occasional invasion of the cancer cells into the dilated lymph vessels was observed (Figure 1E, F). The amount of LMVD (25.970 ± 14.9347) in specimens from cases with lymph node metastsis was much higher than those without lymph node metastasis (17.860 ± 6.5640), *p* = 0.015 (Figure 3A).

LMVD was also observed to be linked to P + V+. The amount of LMVD was 24.727 ± 13.772 in specimens with P + V+, 19.860 ± 6.663 in P + V-, 20.395 ± 10.137 in P-V+, and 13.453 ± 4.503 in P-V-. Compared with other three groups, LMVD in P + V + was significantly increased, *p* = 0.004 (Figure 3B).

### Prognostic significance of PDGF-BB and VEGF-C coexpression in primary human NSCLC

**Figure 4:**
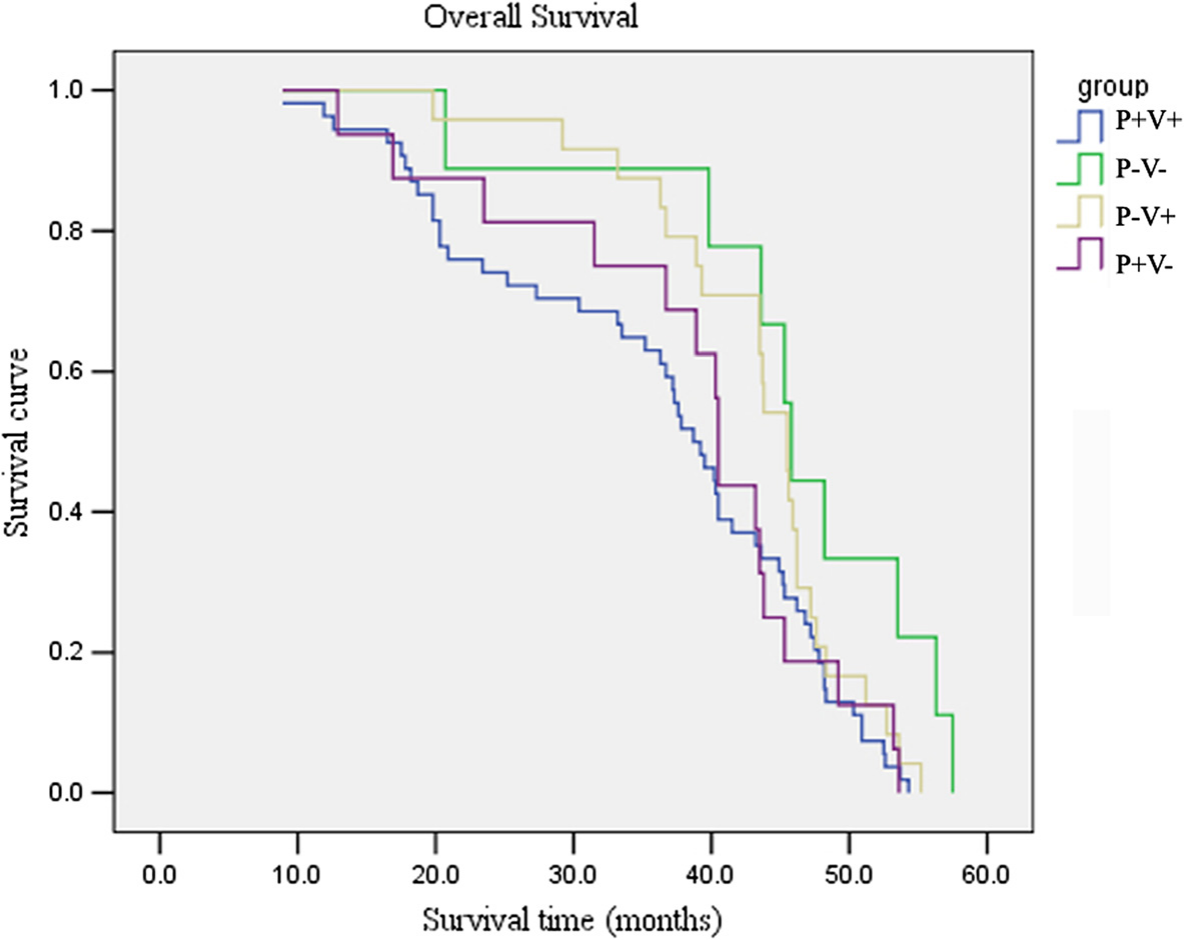
Relationship between coexpression of VEGF-C and PDGF-BB and overall survival in primary NSCLC patients.

P + V + was correlated with poor overall survival (OS). The univariate survival analysis showed that, cases with P + V + had shorter survival time (38.7 m ) compared with those with P-V- (45.8 m), *p* = 0.015. However, no significant relationship was observed between OS and P + V- or P-V + ( Figure 4).

### Disscussion

Today accumulating evidences show that tumor may establish not only their own new blood vessels supply, but might also induce lymphangiogenesis to promote its spread [29]. So possible inhibition of those processes might be of benefit for cancer patients, especially as recent data suggest that the process of lymphangiogenesis is not only limited to primary tumor, but is also present in lymph node metastases, resulting in further cancer cell spread [30]. In this study, we found the disordered and dilated lymphatic vessels were almost exclusively in peri-tumoral lesions but not in intra-tumoral lesions. And the amount of LMVD in cases with lymph node metastasis was significantly higher than those without lymph node metastasis. The results showed lymphangiogenesis existed in NSCLC tissues and was associated with lymphatic metastasis, which is consistent with previous reports [11], and might be explained by a rising interstitial pressure caused by an increase in the size of lesion or by the lack of intratumoral lymphangiogenesis in NSCLC [31]. Indicating that peri-tumoral lymphatic vessels are important for the process of metastatic spread while intra-tumoral lymphatic vessels are non-functional [32,33].

Lymphangiogenesis may require the interaction of several tumor-derived growth factors. It is demonstrated that VEGF-C and PDGF-BB are both important growth factors contributing to lymphangiogenesis [22]. VEGF-C can activate the VEGFR-3 signaling pathway to induce the lymphatic enlargement and lymphangiogenesis [14]. A study demonstrated that PDGF-BB can promote lymphangiogenesis and lymphatic metastasis by a VEGFR-3 independent mechanism in the mouse cornea in vivo [19]. In this model, the lymphangiogenesis induced by PDGF-BB could not be restricted by blocking interaction of VEGF-C with VEGFR-3, suggesting that, PDGF-BB exerts its effect via an independent pathway that may involve PDGF receptors on lymphatic vessels [34]. Another study showed that VEGF-C is an essential regulator determining PDGF-BB expression for vascular stabilization via a paracrine mode of action [22]. The stimulation of proliferation of lymphatic endothelial cells by platelets seems to be induced in a time and dose dependent manner mainly by VEGF-C and PDGF-BB, which are secreted by platelets. Blocking the experiments indicate a predominant role of VEGF-C in this process [35]. All those results suggested that both factors play complicated roles in tumor lymphangiogenesis. However, the overlapping biological effects of these two factors have not been clarified clearly in human cancers. In this study, overexpression of both PDGF-BB and VEGF-C significantly correlated.

LMVD. Those cases were also younger and had larger tumor size, more likely lymph node metastasis, worse histological differentiation and poorer OS. In addition, a significant association between VEGF-C overexpression alone and worse histological differentiation was found. For the rest, however, PDGF-BB or VEGF-C alone was not linked to any other clinical feature including LMVD. The results indicated NSCLC patients who had overexpression of both PDGF-BB and VEGF-C might present with more rapid growth and higher potential for invasion due to their lymphangiogenesis. Thereby these patients had poorer OS, which was consistent with the results in patients with esophageal squamous cell carcinoma, those with positive expressions of PDGF-BB and VEGF-C have been shown to possess a worse prognosis, compared to those with negative expressions [23]. Also, those results suggested that poorly differentiated cancer cells might be more capable to secrete VEGF-C and PDGF-BB, which induced lymphangiogenesis, thereby promoting disease progression in NSCLC.

The secretion of VEGF-C or PDGF-BB by tumor could induce the activation of their receptors on the vascular endothelium and thereby inducing the formation of new lymphatic vessels [36]. However, little is currently known about the interplay among these lymphangiogenic factors. In this study, a significant positive correlation between PDGF-BB and VEGF-C protein expression of tumor cells was seen in NSCLC, suggesting a lymphangiogenesis pathway that one factor (PDGF-BB or VEGF-C alone) may up-regulate the other factor expression in the same cells. Therefore, we suspected that PDGF-BB and VEGF-C could synergistically promote NSCLC lymphangiogenesis, and enhance the tumor growth and lymph node metastasis. Combined targeting both PDGF-BB and VEGF-C may become a promising strategy for the treatment of NSCLC.

## Conclusions

We found for the first time that compared with the overexpression of PDGF-BB or VEGF-C alone, both PDGF-BB and VEGF-C overexpression in primary human NSCLC was significantly associated with lymphangiogensis and poor outcome. Furthermore, our data suggested that PDGF-BB and VEGF-C expression might have a correlative dependence and interplay, not only in NSCLC lymphangiogenesis, but also in cancer progression. Based on the expression of PDGF-BB and VEGF-C, we speculated the therapy targeting VEGF-C expression in combination with targeting PDGF-BB might be an important approach for control the cancer growth in patients with NSCLC having high expression of both PDGF-BB and VEGF-C.

## Competing interests

All authors declare they have no actual or potential competing financial interests.

## Authors’ contributions

All authors read and approved the final manuscript. JL and CL designed the study, analyzed the data and drafted the manuscript. LQ and JL assisted with the design of the study and collected clinical data. JL and PZ carried out the immunohistochemi- stry and collected clinical data. YS conceived and designed the study, analyzed the data and edited the manuscript.
